# Selection for amoxicillin-, doxycycline-, and enrofloxacin-resistant *Escherichia coli* at concentrations lower than the ECOFF in broiler-derived cecal fermentations

**DOI:** 10.1128/spectrum.00970-24

**Published:** 2024-09-13

**Authors:** Aram F. Swinkels, Egil A. J. Fischer, Lisa Korving, Nina E. Kusters, Jaap A. Wagenaar, Aldert L. Zomer

**Affiliations:** 1 Division of Infectious Diseases and Immunology, Faculty of Veterinary Medicine, Utrecht University, Utrecht, the Netherlands; 2 Department of Population Health Sciences, Faculty of Veterinary Medicine, Utrecht University, Utrecht, the Netherlands; 3 Wageningen Bioveterinary Research, Lelystad, the Netherlands; 4 WHO Collaborating Centre for Reference and Research on Campylobacter and Antimicrobial Resistance from a One Health Perspective/WOAH Reference Laboratory for Campylobacteriosis, Utrecht, the Netherlands; University of Torino, Turin, Turin, Italy

**Keywords:** antimicrobial resistance, minimal selective concentration, *E. coli*, poultry, antimicrobial compounds, resistance genes

## Abstract

Antimicrobial resistance (AMR) is an emerging worldwide problem and a health threat for humans and animals. Antimicrobial usage in human and animal medicine or in agriculture results in selection for AMR. The selective concentration of antimicrobial compounds can be lower than the minimum inhibitory concentration and differs between environments, which can be a reason for bacterial resistance. Therefore, knowledge of the minimal selective concentration (MSC), under natural conditions, is essential to understand the selective window of bacteria when exposed to residual antimicrobials. In this study, we estimated the MSCs of three antimicrobials, amoxicillin, doxycycline, and enrofloxacin in a complex microbial community by conducting fermentation assays with cecal material derived from broilers. We examined the phenotypic resistance of *Escherichia coli*, resistome, and microbiome after 6 and 30 hours of fermenting in the presence of the antimicrobials of interest. The concentrations were estimated to be 10–100 times lower than the epidemiological cut-off values in *E. coli* for the respective antimicrobials as determined by EUCAST, resulting in an MSC between 0.08 and 0.8 mg/L for amoxicillin, 0.4 and 4 mg/L for doxycycline, and 0.0125 and 0.125 mg/L for enrofloxacin. Additionally, resistome analysis provided an MSC for doxycycline between 0.4 and 4 mg/L, but amoxicillin and enrofloxacin exposure did not induce a significant difference. Our findings indicate at which concentrations there is still selection for antimicrobial-resistant bacteria. This knowledge can be used to manage the risk of the emergence of antimicrobial-resistant bacteria.

**IMPORTANCE:**

Antimicrobial resistance possibly affects human and animal health, as well as economic prosperity in the future. The rise of antimicrobial-resistant bacteria is a consequence of using antimicrobial compounds in humans and animals selecting for antimicrobial-resistant bacteria. Concentrations reached during treatment are known to be selective for resistant bacteria. However, at which concentrations residues are still selective is important, especially for antimicrobial compounds that remain in the environment at low concentrations. The data in this paper might inform decisions regarding guidelines and regulations for the use of specific antimicrobials. In this study, we are providing these minimal selective concentrations for amoxicillin, doxycycline, and enrofloxacin in complex environments.

## INTRODUCTION

It has been estimated that in 2050 more deaths will be caused by antimicrobial-resistant bacteria than cancer if no measures are taken (1). This is mainly driven by the use of antimicrobials in human medicine and in agriculture (2). The discovery of new antimicrobial compounds is progressing slowly, which is problematic as they are crucial for modern medicine (2, 3). Despite all efforts to decrease antimicrobial usage, it still remains a problematic issue; in that light, it is important to continue research on how and when the selection of antimicrobial-resistant bacteria takes place. An understudied field in antimicrobial resistance (AMR) research is the effects of antimicrobial residues that remain in the environment long after their application.

In livestock, antimicrobial treatment lasts several days followed by a withdrawal period to ensure antimicrobial compounds do not exceed the maximum residue limit (4, 5). However, some antimicrobials are able to remain longer in the animal or farm environment due to stable chemical properties (6), potentially resulting in an extended period of selection for antimicrobial-resistant bacteria. In pharmacodynamic models, it is generally assumed that selection of antimicrobial-resistant bacteria by an antimicrobial only occurs between the minimum inhibitory concentration (MIC) of a susceptible bacterium and the MIC of a resistant bacterium due to the selective pressure of the antimicrobial compound (7, 8). Gulberg et al. described that sub-MICs, concentrations ranging between the minimal selective concentration (MSC) and the MIC of susceptible bacteria, can also select for antimicrobial-resistant bacteria (9, 10). The MSC is the lowest concentration that favors the growth of antimicrobial-resistant bacteria over antimicrobial-susceptible bacteria (10). Below the MSC, susceptible bacteria can outcompete antimicrobial-resistant bacteria as a consequence of fitness cost by harboring a resistance gene or mutations (11).

Selection for antimicrobial-resistant bacteria occurs mostly in the gastrointestinal tract as conditions are favorable for the outgrowth of resistant bacteria due to conditions that promote bacterial growth and niche competition (12, 13). Moreover, the high density of bacteria facilitates horizontal gene transfer between organisms in the gut microbiome as well as the increased chance of *de novo* resistance mutations because of the high cell densities (1, 14). Therefore, the concentration of antimicrobials reaching the gut plays a crucial role in the selection for antimicrobial-resistant bacteria, as resistant bacteria can outcompete susceptible bacteria influenced by the selective pressure of the antimicrobial present at residual concentrations (15). For example, farm animals can excrete the antimicrobial compounds partially or in an unchanged chemical structure, which might result in re-exposure to antimicrobials in the gastrointestinal tract through coprophagic behavior (16). These residual concentrations may be high enough to exceed the MSC and therefore extend selection for resistant organisms long after treatment has ended. Another essential point is that some estimations of the MSC might be 10–100 times lower than the MIC of susceptible bacteria (9). The residual concentrations of a treatment can therefore affect the emergence of AMR, and for that reason, defining the MSC will provide important information.

The MSC has been determined in several other studies; however, they were mostly conducted in rich medium (17). This is in contrast to the gut environment, where nutrients are more scarce as a consequence of poor mixture and the flow through the gut (18). In addition, competition may be fierce between different bacterial species (19). Therefore, it is possible that antimicrobial compounds can select for antimicrobial-resistant bacteria in the gut microbiome at different concentrations. By defining the MSC in non-model organisms using, e.g., resistome or microbiome measurements, it is possible to provide estimates for the selection of resistant bacteria in humans, animals, or the environment.

The aim of this study was to determine the MSC of amoxicillin, doxycycline, and enrofloxacin in a complex microbial community as found in poultry. Therefore, we investigated this by conducting cecal fermentation assays by exposing the cecal contents of broilers to concentrations lower than the epidemiological cut-off values (ECOFF) of the selected antimicrobials to mimic the natural environment in the intestine. Using cecal fermentation, we were able to exactly regulate the concentration to which the cecal contents were exposed. We determined the MSC by analyzing the phenotypic resistance of *Escherichia coli* isolates and determined the microbial composition after exposure to the different antimicrobials to observe a change in the species diversity and presence. In addition to this, the resistome was determined for an increase in resistance genes, which can reveal selective properties of the antimicrobial compound.

## RESULTS

### Phenotypic resistance of *E. coli* isolates from cecal fermentations after exposure to antimicrobials

**Fig 1:**
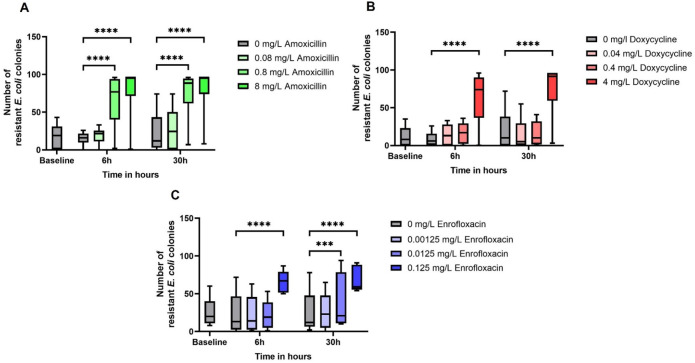
Resistant *E. coli* isolates from the cecal fermentation. In the graphs, the resistant colony count is shown for the different treatments. (A) The amoxicillin treatment, (B) the doxycycline treatment, and (C) the enrofloxacin treatment. In the box and whiskers, the median and the minimal and maximum values are displayed. ***P ≤ 0.001 and ****P ≤ 0.0001.

To investigate the MSC under conditions that mimic the chicken ceca, we exposed cecal contents to different concentrations of antimicrobials and followed the resistance of isolates that are naturally occurring in the ceca. Using cecal fermentations, we are able to exactly titrate the antibiotic concentration, which is impossible in an animal model. Additionally, we sequenced 164 isolates with nine different resistant patterns from our fermentation assays and determined that they belonged to 25 different sequence types (supplemental data), suggesting ample possibility for competition. In the cecal fermentations, we observed a clear shift in terms of resistant *E. coli* isolates for all the treatments, which is shown in Fig. 1. The amoxicillin treatment showed an increase in resistant *E. coli* isolates at 6 and 30 hours when treated with concentrations of 0.8 or 8 mg/L compared to the control group (6 hours, 0.8 mg/L, *P* < 0.0001; 30 hours, 0.8 mg/L, *P* < 0.0001; 6 hours, 8 mg/L, *P* < 0.0001; and 30 hours, 8 mg/L, *P* < 0.0001). This indicates that the MSC of amoxicillin for *E. coli* ranges between 0.08 and 0.8 mg/L. The treatment with doxycycline at a concentration of 4 mg/L at time points 6 and 30 hours resulted in an increase in resistant *E. coli* compared to the control group (6 hours, 4 mg/L, *P* < 0.0001; 30 hours, 4 mg/L, *P* < 0.0001). The treatment with 0.4 mg/L doxycycline showed a significant value at 6 hours. However, we did not show this point in the graph since some experiments had a few to zero doxycycline-resistant *E. coli* isolates.

We performed the statistical tests without the experiments with a few or zero doxycycline-resistant *E. coli,* which resulted in a non-significant difference between the 6 hours, 0.4 mg/L doxycycline sample and the control group at 6 hours in contrast to the treatments with 4 mg/L doxycycline. The MSC of doxycycline for *E. coli* is therefore estimated between 0.4 and 4 mg/L. Finally, we observed an increase in the resistant *E. coli* isolates at a concentration of 0.125 mg/L enrofloxacin at time points 6 and 30 hours (6 hours, 0.125 mg/L, *P* < 0.0001; 30 hours, 0.0125 mg/L, *P* = 0.0005) also at a concentration of 0.0125 mg/L enrofloxacin at 30 hours (*P* < 0.0001). This suggests that the MSC of enrofloxacin for *E. coli* at 6 hours is ranging between 0.0125 and 0.125 mg/L. For the treatment at 30 hours, it is estimated between 0.00125 and 0.0125 mg/L.

### Resistome analysis of the cecal fermentations

**Fig 2:**
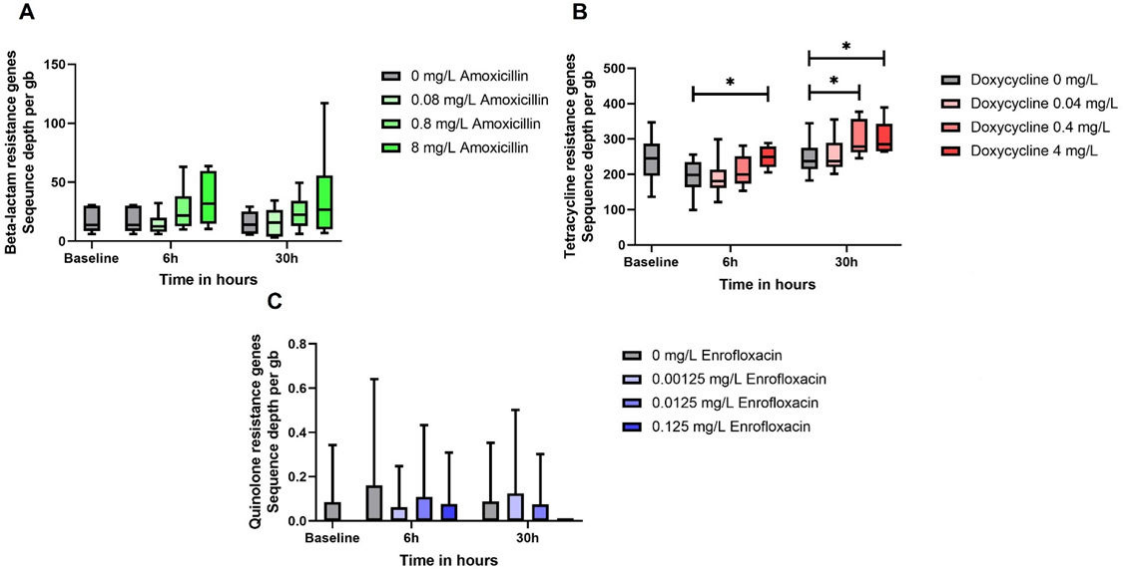
Resistance genes per gigabase in the different treatments. (A) The β-lactam genes in the samples treated with amoxicillin. The graph in panel B presents the tetracycline genes in the samples from the treatment with doxycycline, and in panel C, the graph shows the qnr genes in the treatments with enrofloxacin. In the box and whiskers, the median and the minimal and maximum values are displayed. *P ≤ 0.05

We analyzed the resistome to investigate the effect of treatment with different concentrations on the sequence depth of resistance genes. We considered the specific increase in resistance genes that cause resistance to the antimicrobial used in the cecal fermentation, as shown in Fig. 2.

The treatment with amoxicillin did not show an increase in the sequence depth of β-lactam genes compared to the control group. We did find more variation in the resistance depth of the β-lactam genes per gigabase at the highest concentration of 8 mg/L; however, no significant difference was found. In contrast to the treatment with doxycycline, where the highest concentration of 4 mg/L gave a significant difference with the control groups, both for the 6-hour sample (*P* = 0.037) and the 30-hour sample (*P* = 0.030). In addition, the concentration of 0.4 mg/L doxycycline at time point 30 hours also resulted in a significant difference (*P* = 0.050). We examined the sequence depth of quinolone resistance genes (*qnr*) for the treatments with enrofloxacin, but no differences were found. Resistance to enrofloxacin or fluoroquinolones is mostly mediated by single nucleotide polymorphisms (SNPs) in the quinolone resistance-determining regions (QRDR) of the *gyrA* and *parC* genes that play a crucial role in DNA replication (20). However, we were unable to determine the difference in SNPs between the control treatments and enrofloxacin in the metagenomic data as no software currently exists for SNP-based analysis of resistance in metagenomes.

### Microbiome composition

The microbiome was analyzed to study the effect of the antimicrobial treatments on the microbial composition. We studied the effect of treatment on the alpha- and beta-diversity of the fermentations. Alpha-diversity was determined by the Shannon index, and the beta-diversity was estimated by the Bray-Curtis distance from the baseline measurement (*T* = 0) between the different concentrations within a treatment. Additionally, we studied the species composition of the different treatments for a change in the microbial composition.

**Fig 3:**
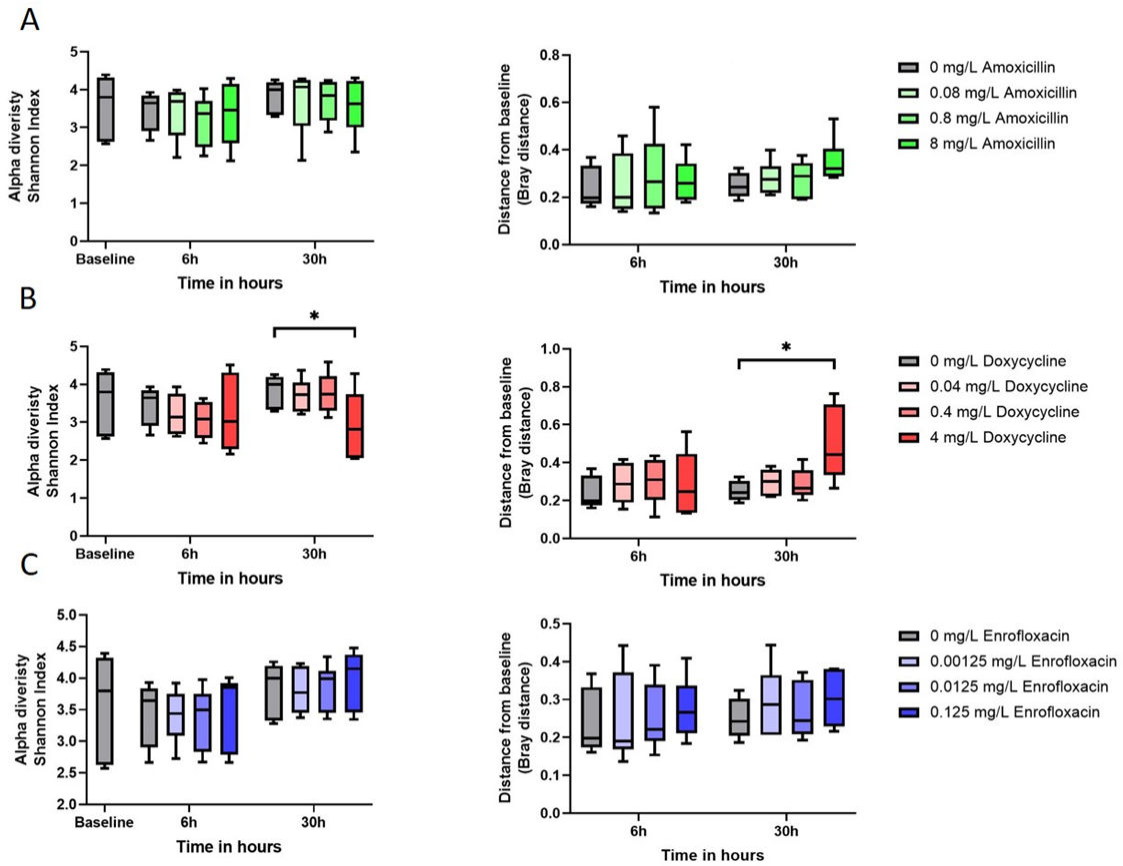
Alpha- and beta-diversity of the different treatments. Alpha-diversity is measured in the Shannon index, and beta-diversity is determined with Bray-Curtis distance. (A) The amoxicillin treatment, (B) the doxycycline treatment, and (C) the treatment with enrofloxacin. In the box and whiskers, the median and the minimal and maximum values are displayed. *P ≤ 0.05.

**Fig 4:**
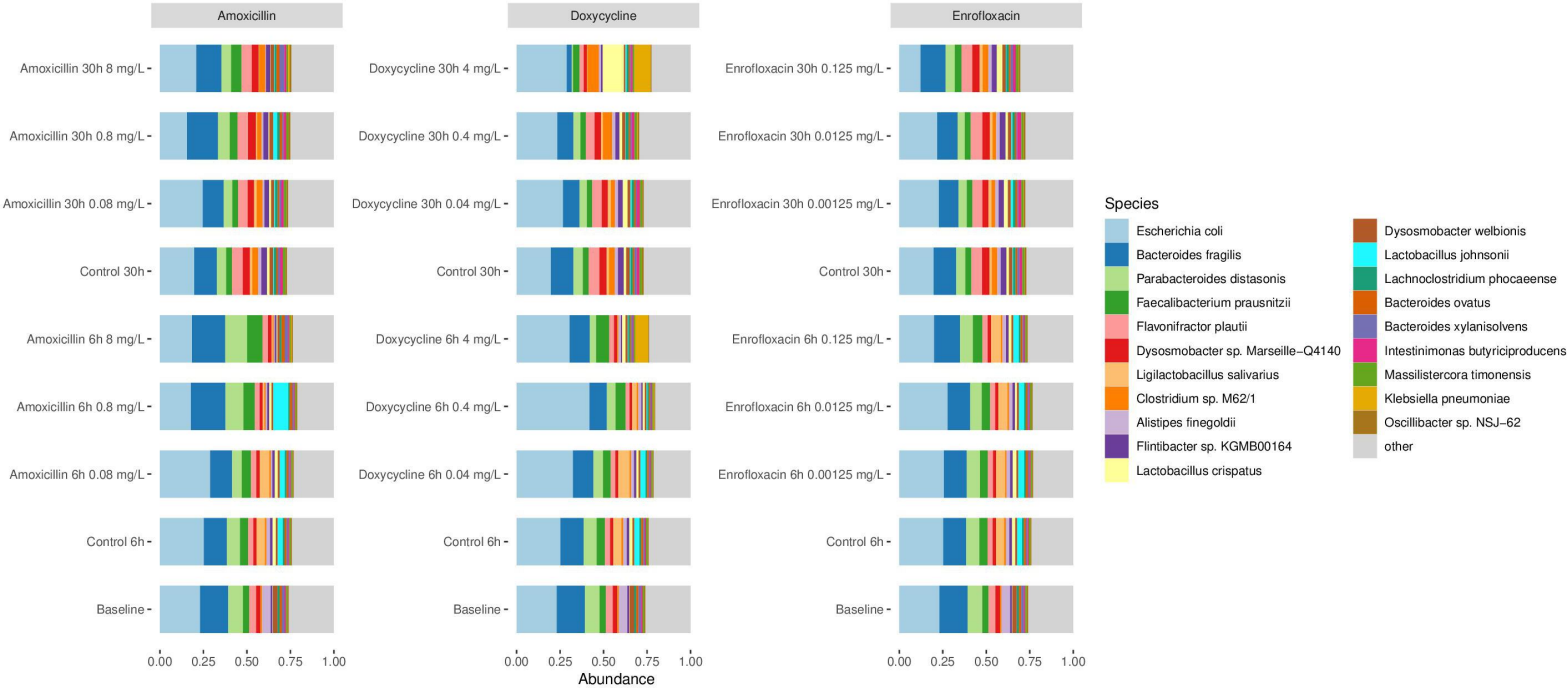
The relative abundance in the cecal fermentations at the species level. The 20 most abundant taxa are shown in the legend on the right side.

The different concentrations of amoxicillin did not show any differences compared to the control group in either alpha-diversity or beta-diversity (Fig. 3). In addition, this was also the observation for the treatment with enrofloxacin. In contrast, the treatment with doxycycline showed a significant difference in alpha-diversity in which the treatment with the concentration of 4 mg/L was different from the control at 30 hours (*P* = 0.023). In addition to this, the beta-diversity was also different between those two treatments at 30 hours (*P* = 0.0053). Thus, the microbial composition was altered by 4 mg/L doxycycline over 30 hours, where we estimated the MSC for doxycycline on the microbiome. Besides alpha- and beta-diversity, we studied the microbial composition to observe any alteration, and therefore we also analyzed the relative species abundance in the cecal fermentations, shown in Fig. 4. The species composition differed between the treatments and the control samples. Certain species disappeared under detection level in the microbiota and were replaced by other species. For doxycycline 4 mg/L at 6 and 30 hours, we observed *Klebsiella pneumoniae* abundance, which is in contrast to the control fermentation at the same time points. The enrofloxacin treatment resulted in less abundant *E. coli* compared to the control group especially after 30 hours. The amoxicillin treatment induced mostly a difference in the microbial composition after 6 hours since the composition after 30 hours was comparable to the 30-hour control.

## DISCUSSION

The objective of this study was to determine the MSC values for amoxicillin, doxycycline, and enrofloxacin in a complex microbial community by assessing the fraction of phenotypic resistance of naturally occurring *E. coli* and by measuring the sequence depth of resistance genes using metagenomics sequencing output in cecal fermentation assays in which we exposed the cecal contents of broilers to antimicrobial concentrations lower than the ECOFF. The range of concentrations we used differed by 10-fold steps implying that the MSC could range between the MSC determined and one concentration below that. Therefore, we display the MSCs as a range between two values.

We were able to distinguish an increase in the resistant *E. coli* isolates compared to the control groups per treatment. The MSCs were determined for amoxicillin from 0.08 to 0.8 mg/L, doxycycline 0.4–4 mg/L, and enrofloxacin 0.0125–0.125 mg/L. AMR of *E. coli* is positively correlated with the AMR in the bacterial population (21, 22). The comparison of the MSC observed under defined conditions in the lab versus in a complex environment is not as straightforward. Klümper et al. (23) showed that the MSC in a complex environment is higher, which is comparable to our results (23). This counterintuitive result was explained by enhanced resistance spread in a complex microbial community, as originally one could expect the MSC to be lower because of increased strain competition between species due to increased fitness costs of resistance. We suspect that antimicrobial drug degradation may also increase the MSC.

Comparing the MSC in a complex environment to the ECOFF is not straightforward, the ECOFF is determined at the population level and distinguishes wild-type bacteria from non-wild-type bacteria, even though the same selective properties in complex environments may be responsible for the observed phenomena. The actual resistance levels measured, as defined by the MIC value, take place under laboratory conditions from which the ECOFF is derived. Therefore, we compared the MSC values we determined to the ECOFF values from EUCAST (24). To elaborate, the MSC of amoxicillin determined for *E. coli* was between 0.08 and 0.8 mg/L, which is almost 10–100 times lower compared to the ECOFF, which has been determined at 8 mg/L. The MSCs of *E. coli* determined in our study for doxycycline (0.4–4 mg/L) and enrofloxacin (0.0125–0.125 mg/L) are 0–10 times lower compared to the ECOFFs [doxycycline 4 mg/L (since 27 February 2023 8 mg/L) and enrofloxacin 0.125 mg/L] of EUCAST (24). Selective concentrations can be up to 100 times lower than the ECOFF and still select for resistant *E. coli*. Nevertheless, *E. coli* represents only a part of the microbiome, making it imperative to investigate the resistance genes in the resistome in order to include other species.

For this reason, the resistome was determined in the cecal fermentations, which showed different results compared to the phenotypic resistance analysis. We found an increase in the tetracycline resistance genes in the doxycycline treatment at concentrations between 0.4 and 4 mg/L after 6 hours, and after 30 hours, it was decreased to 0.04 and 0.4 mg/L. This was 10-fold lower than the MSC determined by phenotypic resistance for *E. coli*. This can be due to the increased pool of genes when determining the resistome compared to solely *E. coli*. For the amoxicillin treatment, we observed an increase at the 8 mg/L amoxicillin after 30 hours; however, this increase was probably a consequence of extensive variation due to the repeated experiments since no significance was found. A reason for this could be a result of the poor stability of amoxicillin (6). Amoxicillin is temperature sensitive and as we incubated the cecal fermentations at 41°C, its stability might have decreased during the fermentation (25). In addition, the existing resistance could also influence the effectiveness of amoxicillin since β-lactamase is able to hydrolyze the β-lactam ring and therefore inactivate the compound (26). Similarly, we did not observe any increase in the sequence depth of quinolone resistance genes in the enrofloxacin treatments, but this is assumed to be caused by the resistant mutation-selection mechanism in the QRDR region in the form of point mutations (20), which are not picked up by measuring sequencing depths of transferable resistance genes.

Antimicrobial compounds can also induce a change in the microbial composition due to its selective properties (27). In the treatments with amoxicillin and enrofloxacin, we did not observe alterations in the alpha- or beta-diversity, which conflicts with a previous study where higher concentrations of amoxicillin (11 mg/kg of body weight) and enrofloxacin (5 mg/kg of body weight) were used (28). Another reason could be that the selection of bacteria in the microbiome is driven by the presence of resistance genes in the pool of genes, which may be much larger in farms as whole flocks of broilers are treated. Residual antimicrobial concentrations will select for bacteria in the composition that express resistance genes for that specific antimicrobial compound (29). Treatment with doxycycline 4 mg/L at 30 hours showed significant differences, similar to studies with doxycycline (100 mg/mL per kg body weight) and chlortetracycline (2 g/L), albeit these concentrations were considerably higher than the concentrations used in our study (30, 31). The significant observation at 30 hours could be attributed to the time the microbiome needs to grow by binary fission as only a proportion of the initial microbiome grows when adding the antimicrobial (27, 32).

Another essential point could be the selective properties of the VL medium that has been used to cultivate the bacteria. It is not unlikely that not all bacterial species will survive in the VL medium as bacteria have unique growth conditions, thus selection for a number of species occurs which might influence the diversity (33). The cecum contains a mixture of anaerobic and facultative anaerobic bacteria (34, 35), and the transfer of the cecum material to the Eppendorf tubes for fermentation could have a selective effect. Strict anaerobes may not have survived the brief exposure to oxygen during transport from the dissecting table to the anaerobic hood and this might have affected the diversity. Alteration, reduction, or selection of species in the microbiome after an antimicrobial treatment have been demonstrated in the literature, as we have also observed (29, 36).

The conditions we used in the cecal fermentations have been set to mimic the environment in the intestine as best as possible and therefore generated, to our belief, MSCs that are applicable to the physiological situation under controlled conditions. Despite the optimal conditions we implemented in this study, fecal fermentation lacks some factors compared with the natural gut environment. For example, external factors are not taken into consideration, such as the transmission of microbes via litter uptake and fluctuating abiotic conditions such as temperature, or as previously mentioned, selection through the medium (33, 37). Furthermore, each repetition is from two different broilers originating from different flocks. It was unknown if the broiler flocks had high or low pre-existing resistance due to farm practices or earlier treatments. As a consequence, each repetition has different microbial flora and resistant gene pools due to the environment the broilers encountered. This is clearly visible in our data in which we observed variation at some time points of the treatments. For instance, in the phenotypic resistance assays of *E. coli*, resistome analysis of the amoxicillin treatment, or the diversity of the enrofloxacin treatment. Interestingly, the MSC remains fairly constant. In previous studies, the MSC has been determined for a complex microbial community, but in contrasting study designs that also generated MSCs for ciprofloxacin and tetracycline, which are comparable to enrofloxacin and doxycycline (37, 38). In these studies, phenotypic resistance of the present bacteria and shotgun analysis for the resistome were also performed. The MSC provided by Lundström et al. (37) of tetracycline ranges from 1 to 10 μg/L. By phenotypic analysis, the MSC was determined at 10 µg/L and by examination of the resistance genes of *tetA* and *tetG* at 1 µg/L. The MSC determined here is extensively lower than the ECOFF of 8 mg/L provided by EUCAST, approximately 800 times (24, 37), while the MSC we provided in our study for doxycycline was equal or up to 10 times smaller than the ECOFF. On the other hand, Stanton et al. (38) also examined the MSC for ciprofloxacin by quantifying the *intI1* integrase at different concentrations at which they found an MSC at approximately 10.77 µg /L, which is comparable for the MSC we determined for enrofloxacin ranging from 0.0125 to 0.125 mg/L. Although the authors of these papers also investigated the MSC in a complex microbial community, one significant difference is that their samples were obtained from wastewater plants. Especially the difference in the MSC of tetracycline and doxycycline could be explained by the difference in the composition of the wastewater, which is a collection of feces from non-clinical and clinical sources. This could indicate that the pool of resistance genes present is larger at the start, and therefore lower concentrations might already favorably affect the selection for resistant bacteria (39, 40).

The MSCs we have estimated in our research give a better understanding about the selection window for antimicrobial-resistant bacteria in the context of treatment of poultry. Especially in combination with persistent antimicrobial compounds, data on MSCs are valuable to veterinarians and can inform policymakers on how to categorize antimicrobials to reduce the selection for AMR (41, 42). The results gave an insight into the selective concentrations of the antimicrobial compounds used in this study and could therefore contribute to decreasing the threat of AMR.

## MATERIALS AND METHODS

### Sample collection and cecal fermentation

Cecum material was obtained from broilers from commercial farms in the Netherlands, which were culled for welfare reasons as they encountered serious lameness. The broilers ranged in age from 15 to 40 days due to sampling at different times. The experiment was repeated six times, and for each experiment, cecum material of two different broilers was used. The ceca were removed from euthanized broilers, which were used for educational purposes at the Faculty of Veterinary Medicine (registration number: AVD10800202115056). The ceca were transported to the lab, and within half an hour, the contents were obtained from the ceca. Subsequently, the cecal contents of the different ceca were mixed to get a homogenized mixture. Thereafter, 1% inoculation was prepared in 10 mL ViandeLeuvere (VL) medium (43, 44) in a tube and 1 mL was added to an Eppendorf tube. The VL medium was modified by adding cysteine hydrochloride (0.4 g/L), 20% glucose, vitamin K (9 mg/L), hemin (50 mg/L), and bile (5 mg/L) after adjusting the pH to 6.3 and autoclaving. In addition, to buffer the medium, sodium hydrogen phosphate (2.5 mg/L) was added before autoclaving. The fermentations were treated with amoxicillin (8, 0.8, and 0.08 mg/L), doxycycline (4, 0.4, and 0.04 mg/L), enrofloxacin (0.125, 0.0125, and 0.00125 mg/L), and a control treatment was included. The concentrations were selected based on the results of the previously performed enriched competition assay. The fermentation was done in a shaker at 41°C at 300 rpm in an anaerobic hood to mimic the conditions in the intestine. At the beginning of the experiment, a baseline measurement was conducted. Subsequently, after 6 and 30 hour of fermenting, a measurement was taken for every treatment. This experiment was repeated six times, and every experiment was performed on a different day as the ceca from different broilers from different flocks were used. For a detailed description of the cecal fermentation, we included a flowchart present in the supplemental data.

### *E. coli* isolation and phenotypic selection determination

Directly after removing the Eppendorf tubes from the anaerobic hood, 10 µL of each fermentation was inoculated five times on five individual MacConkey plates. After overnight incubation at 37°C, approximately 20 single colonies per MacConkey plate were picked with a pipette tip and transferred to a single well in a 96-well plate that contained 100 µL LB medium per well. The 96 colonies per treatment were transferred to squared MacConkey plates containing ECOFF concentrations of amoxicillin (8 mg/L), doxycycline (4 mg/L), enrofloxacin (0.125 mg/L), cefotaxime (1 mg/L), and a control plate without antimicrobials. Next, resistant colonies were scored after overnight incubation at 37°C. An *E. coli* colony was scored resistant if it was able to grow on the plate with ECOFF concentration of the specific antimicrobial. This allowed us to calculate the number of resistant *E. coli* selected by comparing the growth of the *E. coli* on the control plate with the growth on the selective plates.

### Shotgun metagenomics

DNA from the 72 samples was extracted according to the EFFORT protocol, and the DNA concentrations were measured with a Qubit (45, 46). Next, Illumina sequencing was performed using Illumina NovaSeq 6000 (Useq, Utrecht sequencing facility) with a maximum read length of 2 × 150 bp. Libraries were prepared with Illumina Nextera XT DNA Library Preparation Kit according to the manufacturer’s protocol (47). Afterward, the reads were trimmed with trim galore (version 0.6.4_dev), and the quality was assessed with FastQC (version 0.11.4). Afterward, the reads were analyzed for the taxonomic classification by kraken2, and subsequently, the abundance of the DNA sequences was computed with bracken (48, 49). The output of these packages was summarized into a biomfile using kraken2biom and analyzed with the R program packages Phyloseq (version 1.36.0), Microviz (version 0.9.1), and Microbiome (version 1.14.0), by which the species composition and alpha- and beta-diversity were estimated from a rarefied phyloseq object (50, 51). Finally, the resistome was investigated by using the tool KMA, which aligns reads known to AMR genes using the Resfinder database (52, 53). The reads were first normalized for gene length and displayed as sequence depth per gigabase.

### Bacterial clone sequencing

*E. coli* colonies were selected from the fermentations that have been treated with the highest concentration of the antimicrobials of interest. The selection criteria of the *E. coli* colonies were based on their phenotypic resistance patterns. We isolated *E. coli* colonies (supplemental data) from the fermentations treated with the highest concentrations per antimicrobial compound. Per fermentation, we selected one to six isolates with specific phenotypic resistance patterns. In total, we isolated 164 isolates from all the fermentation experiments. Afterward, we isolated their DNA and performed whole genome sequencing with the Illumina NextSeq500 (Useq, Utrecht sequencing facility) with a maximum read length of 2 × 150 bp, and libraries were created as mentioned for the shotgun metagenomics and reads were also similarly processed. Genomes were assembled using SPAdes version 3.14.1. The quality of the genome assemblies was checked with Checkm version 1.1.3, and only genomes with a contamination threshold of <5% and a completeness threshold of >95% were included in the analysis. Afterward, we performed MLST analysis to determine the different sequence types and Resfinder for genomic resistant analysis (52, 54). The sequence data were uploaded to the SRA under accession PRJEB70300.

### Statistical analysis of the phenotypic resistance *E. coli* assay, resistome, and microbiome of the cecal fermentations

We used a Poisson model for counting data and a linear model for continuous data with the repetitions as random effect, using the glm function in R (4.1.0). We performed a *post hoc* Tukey test to determine whether the treatment groups at the time points 6 and 30 hours were significantly different from their control treatment. The R-script and data files can be found in a zenodo repository (supplemental data).

## Data Availability

The sequence data were uploaded to the SRA under accession PRJEB70300. Supplemental materials for this article may be found online at https://zenodo.org/records/10709126.

## SUPPLEMENTAL MATERIAL

Figure S1 spectrum.00970-24-s0001.pdf Fig. S1: Schematic overview of the cecal fermentation.

Table S1 spectrum.00970-24-s0002.xlsx Table S1: Characteristics of the *E. coli* strains sequenced from the cecal fermentation experiments.

ASM does not own the copyrights to Supplemental Material that may be linked to, or accessed through, an article. The authors have granted ASM a non-exclusive, world-wide license to publish the Supplemental Material files. Please contact the corresponding author directly for reuse.
