# Supplementary material for: Selection for amoxicillin-, doxycycline-, and enrofloxacin-resistant Escherichia coli at concentrations lower than the ECOFF in broiler-derived cecal fermentations
Source: Microbiol Spectr. 2024 Sep 13;12(10):e00970-24. doi: 10.1128/spectrum.00970-24 (PMC11448166; doi:10.1128/spectrum.00970-24)
Supplement: Figure S1 — Fig. S1: Schematic overview of the cecal fermentation. [file spectrum.00970-24-s0001.pdf]

Cecal fermentation

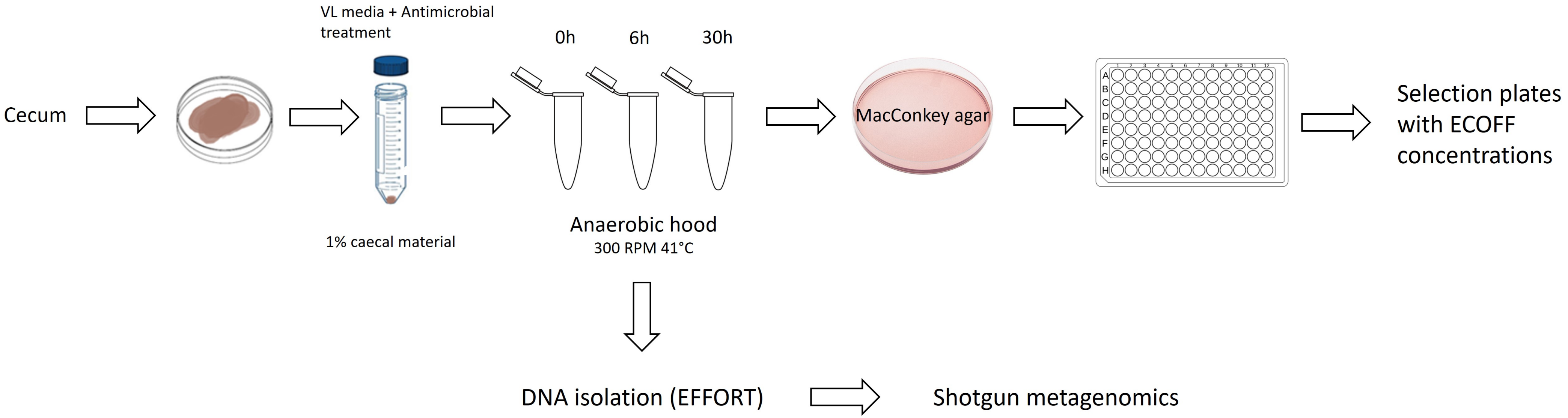

Supplementary Fig S1. Schematic overview of the conduction of the cecal fermentation assay. VL = Viande Levure medium. EFFORT = Ecology from Farm to Fork Of microbial drug Resistance and Transmission'-project.
